# A Study of Feature Combination for Vehicle Detection Based on Image Processing

**DOI:** 10.1155/2014/196251

**Published:** 2014-02-03

**Authors:** Jon Arróspide, Luis Salgado

**Affiliations:** ^1^Grupo de Tratamiento de Imágenes, Universidad Politécnica de Madrid, 28040 Madrid, Spain; ^2^Altran Spain, Methods & Tools, 28022 Madrid, Spain; ^3^Video Processing and Understanding Lab, Universidad Autónoma de Madrid, 28049 Madrid, Spain

## Abstract

Video analytics play a critical role in most recent traffic monitoring and driver assistance systems. In this context, the correct detection and classification of surrounding vehicles through image analysis has been the focus of extensive research in the last years. Most of the pieces of work reported for image-based vehicle verification make use of supervised classification approaches and resort to techniques, such as histograms of oriented gradients (HOG), principal component analysis (PCA), and Gabor filters, among others. Unfortunately, existing approaches are lacking in two respects: first, comparison between methods using a common body of work has not been addressed; second, no study of the combination potentiality of popular features for vehicle classification has been reported. In this study the performance of the different techniques is first reviewed and compared using a common public database. Then, the combination capabilities of these techniques are explored and a methodology is presented for the fusion of classifiers built upon them, taking into account also the vehicle pose. The study unveils the limitations of single-feature based classification and makes clear that fusion of classifiers is highly beneficial for vehicle verification.

## 1. Introduction

Vision-based scene understanding has been the focus of increasing interest for the last couple of decades due to its low cost and flexibility. Among the many fields of application of vision computing, advanced driver assistance systems play a leading role in furtherance of the ambitious goal of reducing car accidents. In particular, studies show that most of the accidents are produced by other vehicles [1]. Therefore, much effort has been devoted in recent years to vision-based vehicle detection.

Most of the video-based vehicle detection methods perform in a two-stage fashion. The first stage addresses hypothesis generation and entails a quick search over the image to find the potential vehicle locations. This stage typically relies on appearance-related features such as color [2], shadow [3], or edges [4]. Then, in the second stage, these hypotheses are further analyzed and a final decision is taken on the presence or not of vehicles in those locations. This paper focuses on the second stage, that is, hypothesis verification.

Vision-based vehicle hypothesis verification methods increasingly resort to learning-based methods, especially on account of the growing processing capabilities. The task is thus usually addressed as a supervised classification problem, in which candidates are classified into one of two classes, that is, vehicles or nonvehicles. In this context, the selection of features to train the classifiers plays a critical role.

Widespread techniques for feature extraction include principal component analysis, wavelet transform, histograms of oriented gradients (HOG), and Gabor filters. Wavelet transform was used in some of the early methods for vehicle verification [5, 6]. The simplest wavelet form is the Haar transform which provides a local analysis of the images, and has been used for feature extraction in many applications, such as image coding, compression, and retrieval. Gabor filters constitute an alternative to wavelets for joint space-frequency representation of images, and have been shown to be better suited for vehicle detection [7]. On the other hand, principal component analysis (PCA) is a well-known technique for feature extraction which has naturally also been used for vehicle images [8, 9]. Finally, histograms of oriented gradients are extensively applied for people detection, in spite of being relatively recent, and are now being explored for vehicle verification (e.g., [10]).

Unfortunately, although these methods are claimed to perform well for vehicle verification, the lack of common databases and of objective and comprehensive tests makes it difficult to have a quantitative measure of the performance of each method for vehicle/nonvehicle classification and of the comparison among them. In addition, although the scarce statistics published for each of them in the literature disclose a reasonably good performance, they also hint that we are still far from flawless classification.

In this context, the combination of the different techniques arises as the natural way to overcome the limitations of each of them and to exploit their heterogeneous nature in a common framework. Unfortunately, the fusion of these techniques has only scarcely been explored in the literature. As an example, in [7], Gabor and wavelet features are combined assuming that they produce complementary results. However, no comprehensive analysis of the fusion potential of feature extraction techniques for vehicle classification is reported in the literature.

**Figure 1:**
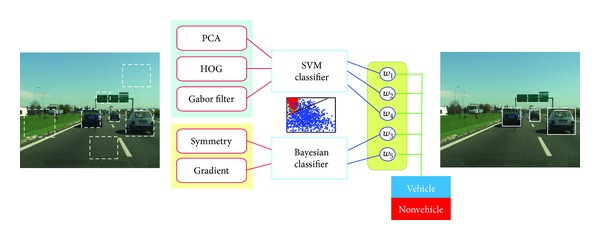
General scheme of the studied fusion approach. A stream of input images is received from a video captured in a traffic scene, together with the potential locations of vehicles (marked through dashed bounding boxes) obtained via an image segmentation technique. The goal is to verify which of them is actually vehicle. To do so, first individual features (PCA, HOG, etc.) are examined, which provide a support for vehicle existence using an associated classifier. Then, a global decision is made according to a classifier fusion scheme.

In this work, the most representative state-of-the-art feature extraction techniques are assessed for vehicle classification, and an in-depth study of the combination of all of them is carried out. First, all these techniques are reviewed and a classification scheme based upon them is presented, which accounts for the pose and distance of the vehicle to the observer. We put a special emphasis on the design of affordable descriptors or the proposal of less-demanding configurations for existing descriptors. In this context, the use of explicit features, which are linked to some *a priori* knowledge on the vehicle appearance, is also explored, in the belief that they can provide fast and meaningful information for the ensemble. In the second part of the paper, a thorough analysis of the fusion capabilities of these techniques is made by considering the diversity of the sources and different normalization and combination procedures. A graphical illustration of the studied fusion approach is provided in Figure 1. Finally, a methodology is proposed to find the best combination of sources according to the vehicle pose. The use of a common public dataset allows us to objectively compare the methods among them and to assess the gain of the fusion approach, which is shown to be substantial.

## 2. Single-Feature Classifiers

According to the revision of the state-of-the-art, in this section, the most successful and representative descriptors are selected and a classification scheme is built upon each of them in order to assess their individual performance and their limits. As stated in Section 1, the most common descriptors are principal component analysis (PCA), histograms of oriented gradients (HOG), and wavelet-based methods, particularly Gabor filters. In this section, the fundamentals of each of these methods are briefly reviewed and descriptors and classifiers based on each of them are presented. Apart from this complex implicit features, the descriptors and classification performance of the most common explicit features, that is, symmetry and gradient, are also enclosed.

A common methodology is employed to evaluate the classification performance of all the descriptors. This relies on a 5-fold 50% holdout cross-validation procedure; that is, for each experiment, half of the samples are randomly selected for the training set and the other half for the testing set. The final performance is measured in terms of the average accuracy (i.e., probability of correct classification) over the five experiments. The database in [11] is used, which is an open access vehicle image database. This dataset is representative in terms of number and variability as it contains 4000 vehicle images (selected to comprise different colors, sizes, vehicle types, etc.) and 4000 nonvehicle images acquired from traffic sequences under different lighting and weather conditions. In addition, vehicles are categorized in four regions (front, left, and right regions in the close/middle range and far range) depending on their relative position with respect to the camera. This will allow for the analysis of different feature combinations according to the vehicle pose.

### 2.1. Principal Component Analysis

The goal of principal component analysis is to derive a smaller set of features which accurately represent the original dataset. In particular, PCA finds the linear subspace of lower dimensionality that maximizes the variance of the original set, which is called principal subspace. A comprehensive description of this method can be found in [12].

Let us denote the data points in the original space **x**_*n*_ in ℝ_*n*_ and their mean and covariance, ***μ***_*x*_ and Σ_*x*_, respectively. As shown in [12], the maximization of the variance is an eigenvalue problem; namely, the principal subspace of dimension *d* is composed of the eigenvectors associated with the *d* largest eigenvalues of Σ_*x*_.

In the case of image feature representation, each image of size *R* × *C* can be represented by a row feature vector *z*_*i*_  (*i* = 1,…, *R* × *C*). According to the above description, the principal subspace is given by the *d* first eigenvectors of Σ_*z*_. Finally, the projections of the original data points onto the directions given by these eigenvectors constitute the PCA features.

Regarding the design of the classifier, support vector machines (SVMs) deliver the best generalization error [13] and are thus also used for the evaluation of implicit feature performance in this work. In particular, a linear SVM is used as a baseline for comparison of the methods.  Table 1 summarizes the performance of such classifier over the PCA features described above for each image region as a function of the principal subspace dimensionality. As can be observed, the optimum dimensionality of the principal subspace varies for the different image regions (40 dimensions for the front close/middle region and 60 for the other regions), and the average detection rate setting the appropriate operation point for each of them is 93.04%.

### 2.2. Histograms of Oriented Gradients

Histograms of oriented gradients describe an image by a dense set of local histograms of gradient orientations. The idea is that the image is divided into disjoint regions, known as cells, and then a histogram is built by counting occurrences of gradient orientations in each region. In practice, the orientation range ([0°, 180°) or [0°, 360°) depending on whether the sign of the gradient is considered) is divided into *β* bins, and each pixel in the region votes for its corresponding bin. In the initial proposal by Dalal and Triggs [14] an additional normalization step is considered in which several adjacent cells are further grouped into blocks so as to relieve illumination and shadowing effects; the histograms of the cells in the block are concatenated and normalized according to the *L*_1_ or *L*_2_ norm. The complete final descriptor comprises the histograms of all the image blocks.

We propose several modifications with respect to the original HOG descriptor in order to better adapt to the addressed multifeature vehicle classification. Indeed, the real-time operation need poses a stringent constraint in the available processing resources and thus in the complexity of the descriptor. Therefore, we propose to adapt to the *a priori* known vehicle structure to modify the descriptor so that it is less demanding but still effective. In particular, vertical and horizontal edges are clearly preeminent in the vehicle rear owing to the rectangular structures in it, such as the back window, the license plate, the taillights, or the vehicle rear contour itself.

**Figure 2:**
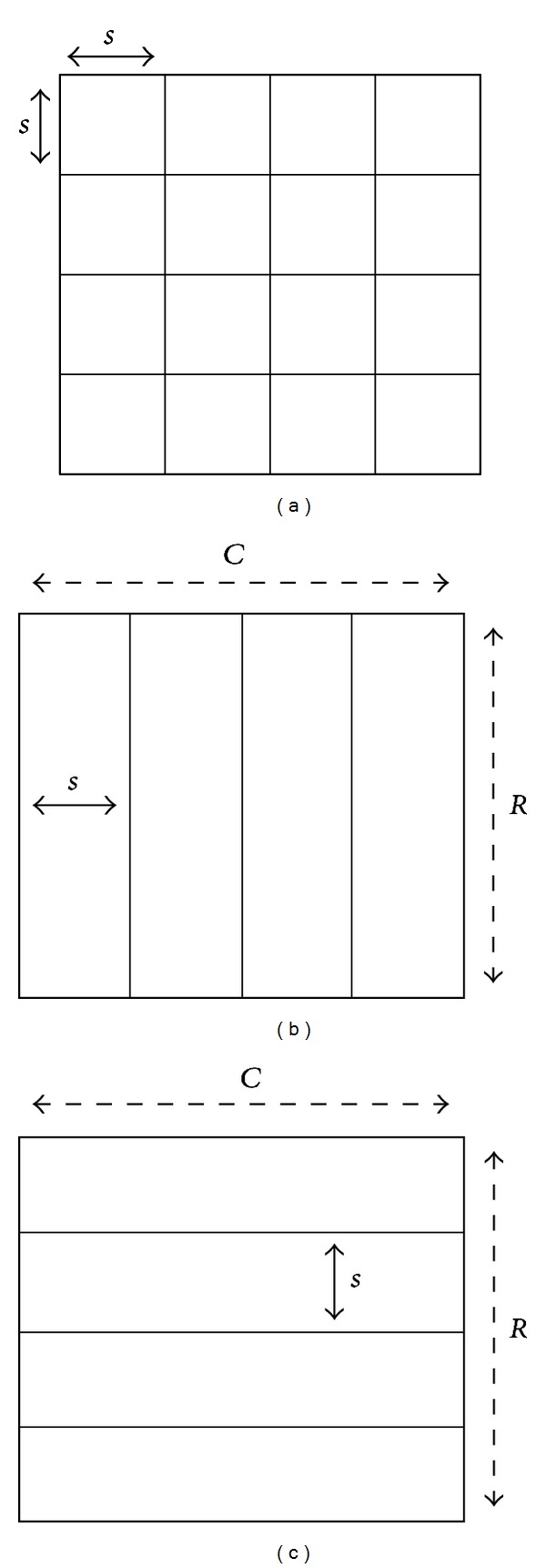
Structure of (a) rectangular HOG as in [14], (b) vertical HOG (V-HOG), and (c) horizontal HOG (H-HOG).

Hence, on the one hand, instead of using a dense grid of square cells as in [14], the image is only divided into vertical or horizontal cells, named, respectively, V-HOG and H-HOG, as shown in Figure 2. An in-depth comparison of V-HOG and H-HOG is performed in [15], where V-HOG is proven to be more efficient than H-HOG (this was also intuitively expected as the frequency of horizontal edges is higher than that of vertical edges, as a result, a larger number of cells is needed in H-HOG). This alternative scheme (V-HOG) involves a processing complexity reduction from *𝒪*(*η* ^2^) to *𝒪*(*η*) with respect to standard HOG, where *η* is the number of cells. On the other hand, the normalization step in [14] entails a big computational overhead, while the performance gain proves to be small for the case of vehicles; hence it has been being dispensed with. In contrast, the sign of the gradient is taken into account as it is informative in some cases.

The performance of the vertical cell HOG descriptor (V-HOG) using SVM is summarized in Table 2. The optimum values of *β* and *η* are selected for each region. As can be observed, the classification accuracy is significantly higher than that of PCA, with an average correct classification rate of 96.46%.

### 2.3. Gabor Filters

Among multiresolution transforms for image processing, Gabor filters display a number of advantages regarding the resolution orientation and aliasing. Hence, they have been broadly used for many applications relating to texture analysis (e.g., [16]) and for image-based object (and in particular vehicle) detection and classification. The spatial 2D Gabor filter is composed of a complex sinusoid carrier and a Gaussian envelope:

(1) g(x,y)=12πσxσyexp⁡{−12[(x−x0)2σx2+(y−y0)2σy2]} ×exp⁡{−2πiF0x}.

The Fourier transform of this Gabor function is thus a Gaussian function shifted from the origin:

(2) $$\begin{array}{c} G\left(u,v\right)=\exp\left\{-\frac{1}{2}\left[\frac{{\left(u-F_{0}\right)}^{2}}{\sigma_{u}^{2}}+\frac{v^{2}}{\sigma_{v}^{2}}\right]\right\}, \end{array}$$

where *σ*_*u*_ = 1/2*πσ*_*x*_ and *σ*_*v*_ = 1/2*πσ*_*y*_ and for simplicity it is assumed that (*x*_0_, *y*_0_) = (0,0).

**Figure 3:**
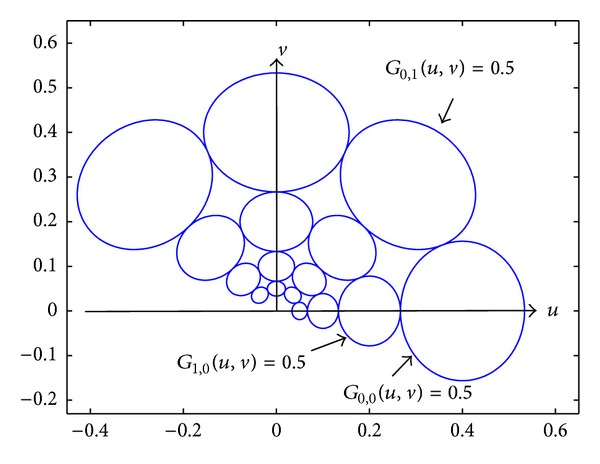
Frequency response of the Gabor filter bank. The contours indicate the half-peak magnitude of the filter responses in the Gabor filter family. The filter parameters used here are *K* = 4, *N* = 4, *a* = 2, and *F*_0_ = 0.4.

In order to capture the frequency content at different scales and orientations, a bank of Gabor filters is required. These filters can be readily obtained by scaling and rotating *g*(*x*, *y*):

(3) gm,n=a−2mg(x′,y′), m=0,…,N−1,n=0,…,K−1,

where *x*′ = *a* ^−*m*^(*x*cos⁡*θ*_*n*_ + *y*sin*θ*_*n*_), *y*′ = *a* ^−*m*^(−*x*sin*θ*_*n*_ + *y*cos⁡*θ*_*n*_), and *a* > 1. The parameters of the bank are thus the number of scales *N*, the number of orientations *K*, the maximum frequency *F*_0_, and the spacing between frequencies, *a*. A graphical representation of the Gabor filter bank in the frequency domain is provided in Figure 3.

In particular, the log-Gabor variation [17] of the Gabor functions is used here. The frequency response of this filter in polar coordinates is

(4) LGm,n(f,θ) ={exp⁡{−(log⁡⁡(f/Fm))22(log⁡βf)2}exp⁡{−(θ−θn)2σθ2}f≠0,0f=0,

where *F*_*m*_ = *a* ^−*m*^ *F*_0_ and *β*_*f*_ and *σ*_*θ*_ represent, respectively, the frequency and angular bandwidth. This family of log-Gabor filters has several advantages over the traditional Gabor functions. In particular, the latter have a nonzero DC-component and therefore provide an excessive overlapping of the filters in the low frequencies. In contrast, log-Gabor filters cover more uniformly the midfrequencies and retrieve the highest frequencies. This is especially important since, as suggested by Field [17], the amplitude of natural images falls of a factor of 1/*f*; thus it has a tail at high frequencies.

An in-depth description of the use of log-Gabor filters for vehicle verification, and proof of its superiority over traditional Gabor filters, is provided in our previous work [18]. As in the prior descriptors, the classification performance is evaluated by means of SVM. The optimum parameters are *N* = 4, *K* = 6, *a* = 2, *β*_*f*_ = 0.65, and *σ*_*θ*_ = 1.5 (see [18]), and the associated accuracy rates are shown in Table 3, together with the values of the maximum frequency, *F*_0_, which varies for the different regions. As can be observed, this descriptor outperforms V-HOG and PCA in the close/middle region but falls below V-HOG in the far range. This unveils the necessity of classifier fusion, as discussed later.

### 2.4. Gradient

Aside from symmetry, gradient has been traditionally the most popular explicit feature for vehicle detection. In a previous work [19], we presented a new simple-but powerful gradient-based descriptor. As opposed to more complex techniques, such as HOG, this descriptor makes use of the knowledge of the vehicle structure in such a way that it achieves high discrimination performance with a small feature set. In particular, two properties relating to vehicles gradient are used: on the one hand, most of the edges are vertical and horizontal, and on the other hand, there is a high density of edges on account of the rich texture in the vehicle rear.

These properties are used in [19] to build a two-feature descriptor based on the HOG scheme. The first feature measures the average distance to the vertical or horizontal direction: the smaller this distance is, the more likely the image corresponds to a vehicle. The second feature is the number of cells with high density of gradients, which discriminates between homogenous background patches (e.g., belonging to the road and the sky) and vehicle instances. Please refer to [19] for more details on this descriptor.

In contrast to the implicit descriptors above, this gradient-based descriptor allows for the use of a generative model. In particular, a bivariate normal distribution is used to fit the data in the described two-feature space and a Bayesian classifier is used to evaluate its performance. Linear and quadratic classifiers are tested assuming, respectively, equal and different covariance matrices for the vehicle and nonvehicle classes. Exhaustive tests for both as a function of the cell size, *s*, and the number of orientations, *β*, can be found in [19]. The results are summarized in Table 4 for the optimum parameters, *s* = 16 and *β* = 18, where it is clear that the quadratic classifier outperforms the linear classifier.

### 2.5. Symmetry

The rear of vehicles is typically symmetrical with respect to the vertical axis. This feature has been widely used in the literature for the detection and classification of vehicles. Most of the reported works make use of the symmetry definition introduced in [20]. In this method, first vertical symmetry is checked for every row of a grayscale image, *I*, by shifting the symmetry axis. If we denote by *x*_*s*_ this symmetry axis and by *u* = *x* − *x*_*s*_, −*w*/2 ≤ *u* ≤ *w*/2 the horizontal shift with respect to it, the symmetry for the row *y*_0_ is

(5) S1D(xs,w,y0) =∫En(u,xs,w,y0)2du−∫O(u,xs,w,y0)2du∫En(u,xs,w,y0)2du+∫O(u,xs,w,y0)2du,

where all possible widths *w* are hypothesized up to the size of *I*, *w* ≤ *C*. In turn, the even and odd parts of *I*, *E*_*n*_, and *O*, in (5), are given by

(6) En(u,xs,w,y0) =E(u,xs,w,y0)−1w∫−w/2w/2E(v,xs,w,y0)dv,E(u,xs,w,y0) ={12(I(xs+u,y0)+I(xs−u,y0)),u∈[−w2,w2],0,otherwise,O(u,xs,w,y0) ={12(I(xs+u,y0)−I(xs−u,y0)),u∈[−w2,w2],0,otherwise.

As stated, the symmetry measure of the input image is computed by integrating 1D symmetry values in the vertical axis, *y*:

(7) $$\begin{array}{c} S_{2D}\left(x_{s},w\right)=\frac{1}{2}\left(\frac{1}{R}\sum_{y=1}^{R}S_{1D}\left(x_{s},w,y\right)+1\right). \end{array}$$

The offset and scaling in (7) ensure that the symmetry value is in the range [0,1], so that it conveys a probability of the image holding a vehicle according to this feature. In particular, the values (*x*_*s*_^*o*^, *w* ^*o*^) maximizing the matrix *S*_2*D*_(*x*_*s*_, *w*) determine the center and bounding box of the hypothesized vehicle.

**Figure 4:**
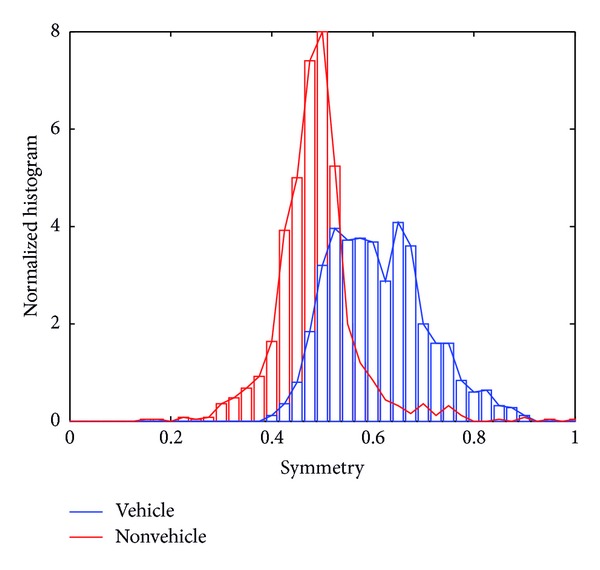
Normalized histogram of symmetry feature for vehicle (blue) and nonvehicle (red) classes in the front close/middle range.

The distribution of this feature for the vehicle and nonvehicle samples in the database is shown in Figure 4 for the front close/middle range. The former is right-skewed and resembles a Rayleigh distribution; this is confirmed by the Kolmogorov-Smirnov test, as shown in [19]. In turn, the nonvehicle distribution is symmetrical and bell-shaped, as the Gaussian distribution, but has heavier tails; thus it is modeled by a *t*-Student distribution. As in the case of the gradient-based descriptor, a Bayesian classifier is used to evaluate the discrimination power of the symmetry feature. This is summarized in Table 5. As expected, the accuracy is more limited than in the previous descriptors due to its very simple nature (as a matter of fact, symmetrical structures may also arise in the background), but it is useful in a multicue approach, as shown later.

## 3. Combination of Classifiers

In the previous sections, a set of descriptors has been analyzed for characterization of vehicles and different classifiers have been presented to address vehicle verification. In this section, we aim to combine the information of the different classifiers so as to enhance the overall recognition performance.

Fusion of information from different sources can be performed at feature level, at matching score level, or at decision level. The latter combines the hard decisions from the different classifiers and is therefore too rigid since much information is lost throughout the classification chain. In turn, although integration at an earlier stage is bound to be more effective due to the richer information of the input data, fusion at feature level is rarely employed in practice as the relation between the feature spaces associated with different information sources is usually unknown. In addition, concatenation of different feature vectors leads to the curse of dimensionality problem [21]. Therefore, a postclassification scheme using the soft outputs of the different classifiers is preferred here.

The goal is thus to combine the output of the different classifiers and to generate a single scalar score. This will be used to make the final decision and also give information on the confidence in the decision. Our ensemble consists of three classifiers based on implicit features, that is, PCA, V-HOG (i.e., the best cost-effective variation of HOG), and log-Gabor, and two classifiers using explicit features, namely, gradient- and symmetry-based classifiers. In addition a classifier ensemble will be designed for each image region, according to the different performance of the above-mentioned classifiers region-wise. However, it must be taken into account that the nature of the output delivered by the classifiers is different. On the one hand, the gradient- and symmetry-based classifiers output likelihoods of the input samples are given the vehicle and the nonvehicle classes, as the distributions of the data have been modeled by known functions (bivariate Gaussian for gradient-based descriptor, Rayleigh, and *t*-Student for symmetry). Since there is no prior information on the classes, *a priori *probabilities are equal and posterior probabilities of each class are just the normalized likelihoods. In contrast, the other three classifiers, based on PCA, HOG, and log-Gabor, are built upon support vector machines and therefore do not provide probabilistic outputs. Instead, a soft value *y* is output that measures the distance to the decision surface, *y* = 0: if *y* ≤ 0, the sample is classified as vehicle, if *y* > 0 as nonvehicle. Hence, a normalization scheme is necessary that transforms these values to a common range [0,1] indicating the support for the hypothesis that the input vector submitted for classification comes from vehicle class. In Section 3.1, the used normalization schemes are described. Once the classifier outputs are in the same domain, normalized scores are combined through a combination rule, as discussed in Section 3.2.

In addition, another key issue for the success of classifier combination is the diversity. Indeed, the classifiers in the ensemble should be as accurate as possible, while at the same time they should not make coincident errors. In other words, we expect that, if one classifier makes errors, there is another classifier that does not make errors in the same input samples (even if it does make errors in others). A number of measures have been proposed for diversity in the literature. Those are reviewed in Section 3.3 and applied to our classifier ensemble.

### 3.1. Normalization of Classifier Outputs

The objective of normalization is to have the output of the classifiers in the same range, so that fusion can be performed. As stated, the classifiers using explicit features, that is, those based on symmetry and gradient, deliver likelihoods of the samples to belong to each of the two classes, *p*(*x*_*i*_ | *V*) and *p*(*x*_*i*_ | *N*), where *V* indicates the vehicle class and *N* indicates the nonvehicle class. Since prior probabilities of vehicle and nonvehicle classes are equal, *p*(*V*) = *p*(*N*) = 0.5, the posterior probabilities are given by

(8) $$\begin{array}{c} P\left(V\;|\;x_{i}\right)=\frac{p\left(x_{i}\;|\;V\right)}{p\left(x_{i}\;|\;V\right)+p\left(x_{i}\;|\;N\right)},\\ P\left(N\;|\;x_{i}\right)=1-P\left(V\;|\;x_{i}\right). \end{array}$$

In particular, we will only retain the probability *P*(*V* | *x*_*i*_). On the other hand, a normalization rule is sought that transforms the soft output *y* of SVM to the range [0,1], indicating the support of the vehicle class. Several normalization schemes have been proposed in the literature, such as min–max, *z*-score, tanh, or double sigmoid normalization (see [21] for a complete survey). In particular, min–max normalization is extensively used [22]. Although this technique is very efficient, it also lacks robustness to outliers; therefore its variant robust min–max is sometimes preferred [23]. In this study, the most popular methods, that is, robust min–max and double sigmoid normalization, are adopted and compared. These rules are described below, where the normalized output is denoted by $\bar{y}$.

**Figure 5:**
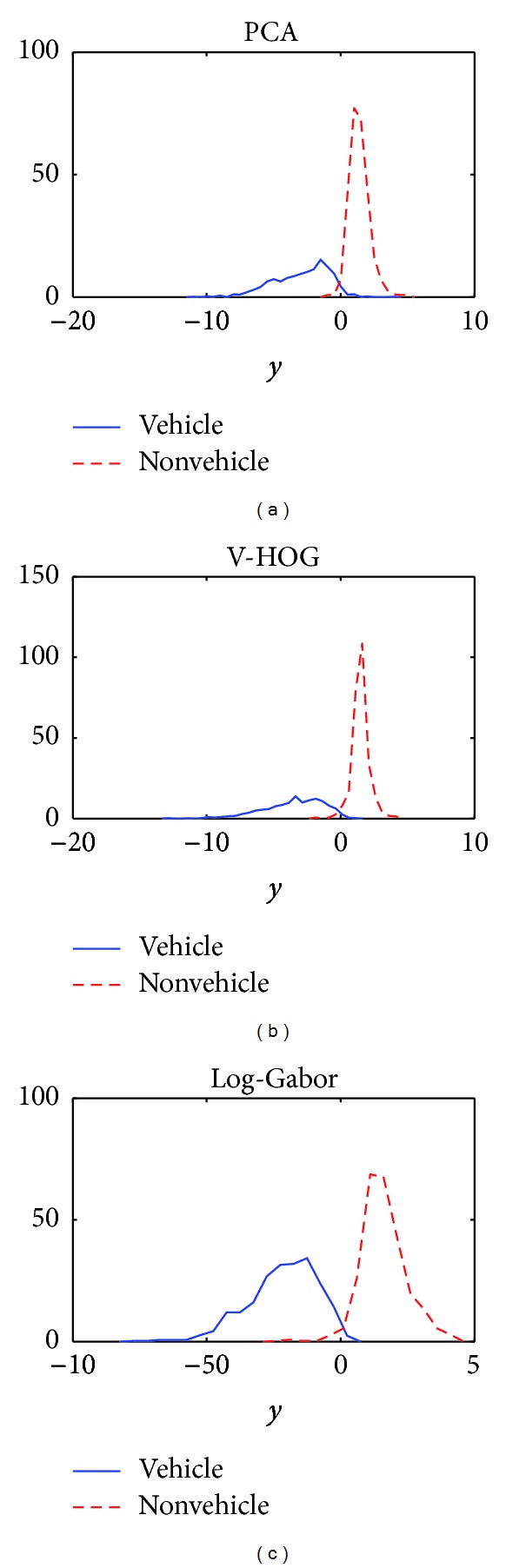
Distribution of soft output delivered by SVM for PCA, V-HOG, and log-Gabor based classifiers.

(i) Min–max:
  (9) $$\begin{array}{c} \bar{y}=\frac{y-y_{\min}}{y_{\max}-y_{\min}}, \end{array}$$

where *y*_min⁡_ and *y*_max⁡_ denote, respectively, the minimum and maximum values of the SVM classifier for the dataset. This normalization transforms the values to the [0,1] range while maintaining their original distribution. As *y*_min⁡_ and *y*_max⁡_ are extracted from the dataset, this method is highly sensitive to outliers and robust min–max is preferred.

(ii) Robust min–max: it is similar to min–max, only the *y*_min⁡_ and *y*_max⁡_ are selected as the 5 and the 95 percentile of the soft output distribution. As a result, tails are disregarded and the pernicious effect of outliers is avoided. The output distributions for the vehicle (genuine) and the nonvehicle (impostor) classes for the front close/middle region are shown in Figure 5. Soft outputs above zero should be mapped in the interval [0,0.5] and soft values below zero in the interval [0.5,1], indicating negative and positive vehicle support, respectively. Therefore, the normalization rule is
  (10) $$\begin{array}{c} \bar{y}=1-0.5\frac{y-y_{\min}^{g}}{-y_{\min}^{g}}\;\text{for}\;y\leq 0, \end{array}$$
  (11) $$\begin{array}{c} \bar{y}=0.5\frac{y_{\max}^{i}-y}{y_{\max}^{i}}\;\text{for}\;y>0, \end{array}$$

where *y*_min⁡_^*g*^ is the 5 percentile of the genuine class and *y*_max⁡_^*i*^ is the 95 percentile of the impostor class. The parameters (*y*_min⁡_^*g*^, *y*_max⁡_^*i*^) for PCA, V-HOG, and log-Gabor are, respectively, (−6.48,2.73), (−7.58,2.56), and (−4.42,3.19) for the front close/middle region. The values for the remaining regions are derived in the same manner as explained above and are as follows: for the left region, (*y*_min⁡_^*g*^, *y*_max⁡_^*i*^) = (−3.10,3.17), (−7.37,6.99), and (−4.12,6.85); for the right region, (*y*_min⁡_^*g*^, *y*_max⁡_^*i*^) = (−2.68,2.86), (−5.30,5.98), (−5.81,6.00); and for the far region, (*y*_min⁡_^*g*^, *y*_max⁡_^*i*^) = (−2.23,2.52), (−11.20,3.22), (−3.19,4.68).

(iii) Double sigmoid:
  (12) $$\begin{array}{c} \bar{y}=\{\begin{array}{cc} \frac{1}{1+\exp\left(2y/r_{1}\right)}, & \text{if}\;y<0,\\ \frac{1}{1+\exp\left(2y/r_{2}\right)}, & \text{if}\;y>0. \end{array} \end{array}$$

This normalization rule is determined by the values *r*_1_ and *r*_2_, among which the function is linear. In order to set these values, similar to min–max normalization, the 5 percentile of the vehicle class, *y*_min⁡_^*g*^, and the 95 percentile of the nonvehicle class, *y*_max⁡_^*i*^, are retained, and then *r*_1_ = −*y*_min⁡_^*g*^, and *r*_2_ = *y*_max⁡_^*i*^. This way, $\bar{y}=1/(1+\exp(-2))≃0.88$, for *y* = *y*_min⁡_^*g*^ and the support decreases linearly to $\bar{y}=1/(1+\exp(2))≃0.12$ for *y* = *y*_max⁡_^*i*^. The tails, in contrast, decrease nonlinearly.

### 3.2. Combination Rules

Let **x** ∈ ℝ^*n*^ denote an input sample and let *𝒟* = {*D*_1_, *D*_*n*_,…, *D*_*L*_}, *L* = 5, be the set of classifiers. As a result of normalization, each classifier delivers a value in the interval [0,1]; that is, *D*_*i*_ : ℝ^*n*^ → [0,1]. This value is the support that classifier *D*_*i*_ gives to the hypothesis that **x** corresponds to the vehicle class, denoted by *d*_*i*_(**x**). The overall degree of support, *μ*(**x**), is a combination of the individual supports given by the classifiers. Among the several combiners proposed in the literature (see [24], chapter 5, for an exhaustive survey), we adopt the most popular ones, that is, simple average and weighted average.

(i) Simple average:
  (13) $$\begin{array}{c} \mu \left(x\right)=\frac{1}{L}\sum_{i=1}^{L}d_{i}\left(x\right). \end{array}$$
(ii) Weighted average:
  (14) $$\begin{array}{c} \mu \left(x\right)=\sum_{i=1}^{L}w_{i}d_{i}\left(x\right). \end{array}$$

The weights are typically selected to minimize the variance of *μ*(**x**), with the restriction that ∑_*i*=1_^*L*^ *w*_*i*_ = 1. As shown in [24], one way to find the weights is to assume that the approximation errors, *t*_*i*_ − *d*_*i*_(**x**), are normally distributed with zero mean (in this case, the target value is *t*_*i*_ = 1 for vehicles and *t*_*i*_ = 0 for nonvehicles). Under this assumption, the weights minimizing the variance of *μ*(**x**) are given by [24]

(15) $$\begin{array}{c} w=\Sigma^{-1}I{\left(I^{⊤}\Sigma^{-1}I\right)}^{-1}, \end{array}$$

where **w** = [*w*_1_,…, *w*_*L*_], Σ is the covariance matrix of the classifiers approximation errors, and *I* is a column vector of *L* ones.

### 3.3. Diversity

The success of the ensemble depends to a large extent on the fact that the classifiers complement each other, that is, in the diversity of the classifier outputs. A number of diversity measures have been specifically proposed in the literature for binary output classifiers that classify samples as correct or incorrect (also called oracle output classifiers), both considering the members of the ensemble pairwise and all the classifier ensembles together. The former is adopted here as done in common practice. Popular pairwise diversity measures include the disagreement measure and the Double-Fault measure [24]. Also, more general statistical measures of relationship such as the correlation coefficient and the *Q*-statistic are sometimes used as indicators of the diversity of the ensemble. They all are based in a table of the joint outputs of classifiers *D*_*i*_ and *D*_*j*_, as shown in Table 6. The entries in the table are the probabilities of the respective pair of correct/incorrect outputs. In this work, the Double-Fault measure is used as the main diversity measure as we believe that it is more important to detect the classifiers that commit simultaneous errors than those that are simultaneously correct, especially when the individual classifiers already deliver fairly high correct classification rates, as is the case. In addition, the correlation coefficient and the values of the weights minimizing the variance in the weighted average rule are used as complementary indicators of the diversity whenever the Double-Fault measure is not sufficiently informative. The Double-Fault and correlation measures are defined below.

(i) Double-Fault measure: it gives the probability of both classifiers being wrong. According to Table 6, the measure is given by
  (16) $$\begin{array}{c} {\text{DF}}_{i,j}=d. \end{array}$$
(ii) Correlation coefficient: the correlation between two binary classifier outputs is
  (17) $$\begin{array}{c} \rho_{i,j}=\frac{ad-bc}{\sqrt{\left(a+b\right)\left(c+d\right)\left(a+c\right)\left(b+d\right)}}. \end{array}$$

The strategy for the construction of the ensemble is the following. First, the classifiers are independently trained using the same training set and the performance of the ensemble is evaluated on the testing set according to the combination rules explained in Section 3.2. This is repeated 5-fold using 50% holdout cross-validation, and the average joint performance of the ensemble is derived. Then, a new smaller ensemble is proposed by selecting the two least diverse classifiers and removing the one featuring the worst performance. Then, the overall performance of the new ensemble is evaluated. Only if the overall performance of the ensemble is better (or at least similar), a new iteration is realized by proposing a smaller ensemble using the same strategy. This is repeated iteratively and the smallest ensemble is selected before the joint performance is noticeably degraded. The strategy is carried out independently for the different image regions, as the response of the classifiers varies.

## 4. Results and Performance of Classifier Ensemble

Different classifier ensembles are proposed according to the strategy described above for each image region. The performance of the classifiers is evaluated for the different combinations of normalization and fusion rules explained in Sections 3.1 and 3.2: robust min–max normalization with simple average combination (RMM-SA), robust min–max normalization with weighted average (RMM-WA), double sigmoid normalization with simple average (DS-SA), and double sigmoid normalization with weighted average (DS-WA). The results for the different regions are enclosed in Tables 7–14, including the performance of the proposed ensembles and the diversity measures. As performance is concerned, apart from accuracy, recall and precision rates are also provided for completeness.

### 4.1. Front Close/Middle Region

The ensemble involving all classifiers, based on PCA, V-HOG, log-Gabor (*LG*), symmetry (*S*), and gradient (*G*) features, is almost flawless, as it achieves an overall performance of 99.42% using the double sigmoid normalization and weighted average combination (see Table 7). The weights that decrease the variance of the approximation error are 0.28, 0.15, 0.39, 0.07, and 0.11 for PCA, V-HOG, LG, *S*, and *G*, respectively. The features yielding the largest Double-Fault measure are PCA and symmetry, that is, 9.8 (see Table 8(a)). Hence, the first proposed reduced ensemble removes symmetry, as it is less accurate than PCA and also has the lowest weight of the ensemble. As shown in Table 7, the performance is then degraded in 0.30%, which might not justify the computational saving (only one classifier is omitted). However, note that LG and *G* also feature a DF almost as high and have the largest correlation (see Table 8(b)), so we may well try and also omit *G* as the least accurate classifier among the pair. Observe that the accuracy remains the same as in the previous iteration, thus encouraging the use of the smaller ensemble PCA+V-HOG+LG. In addition, V-HOG and LG entail a high correlation coefficient and have the largest DF, 7.2; thus in the next iteration a reduced ensemble without V-HOG is tested. The accuracy decreases slightly, from 99.12% to 98.94%, as shown in Table 7. However, note that the ensemble composed of PCA and log-Gabor based classifiers involves a performance loss below 0.50%, while the computational saving is significant (only 2 out of the 5 classifiers are used), which may well justify the use of the reduced ensemble.

### 4.2. Left Region

As in the front region, the best joint performance is attained by double sigmoid normalization within a weighted average combination scheme (see Table 9). The weights that decrease the variance of the approximation error in this scheme are 0.31, 0.11, 0.45, 0.09, and 0.04 for PCA, V-HOG, LG, *S*, and *G*. In this case, the greatest *DF* is obtained with the two explicit classifiers (symmetry- and gradient-based). Although the accuracy of *G* is higher than that of *S*, the weight of the former in the final decision is almost negligible, and it also displays a high correlation coefficient of 0.2478 with V-HOG (see Table 10(b)), so we proceed by leaving out gradient-based classifier. Naturally, the results of the reduced ensemble are only slightly worse than those of full ensemble. Among the remaining classifiers, the highest Double-Fault rates are observed for *S*+PCA (13.2) and *S*+V-HOG (13.6); thus one could try and remove symmetry in the next iteration. On the other hand, V-HOG has high correlation with LG and worse performance. Therefore, experiments leaving out either *S* or V-HOG are performed. The ensemble PCA+LG+*S* renders higher accuracy and involves a small loss of 0.14% with respect to the previous iteration ensemble. A further thinning of the ensemble by leaving out symmetry is not worth, as the accuracy falls below 98%. Hence, the ensemble composed of PCA+LG+*S* is held.

### 4.3. Right Region

The full ensemble achieves a performance as high as 98.66% using double sigmoid normalization and weighted average, as shown in Table 11. The weights of the classifiers are 0.34, 0.03, 0.48, 0.10, and 0.06 for PCA, V-HOG, LG, *S*, and *G*, respectively. Since the weight of V-HOG is almost negligible, this is removed in the first iteration. Naturally, the accuracy obtained with the ensemble PCA+LG+*S*+*G* is almost the same. According to Table 12(a), the maximum Double-Fault is committed by *S* and *G*. Although *S* has worse accuracy, *G* has high correlation with LG and lower weight in the ensemble. Experiments confirm that removing *G* yields higher accuracy than leaving out *S* (98.64% versus 98.46%). In fact, the performance of the reduced ensemble PCA+LG+*S* isequal to that of PCA+LG+*S*+*G*. Further reduction of the ensemble has been tested (symmetry is dismissed as PCA and LG only commit 3.80 Double Faults), but the performance decays in 0.32%.

### 4.4. Far Region

The best performance for the full ensemble in the far region is achieved using a robust min–max normalization scheme within a weighted average combination framework. The results of the different reduction iterations in the ensemble are enclosed in Table 13. In this case, the least diverse classifiers are LG and *G*, which have an associated Double Fault of 28 (see Table 14(a)). In the first iteration *G*, which has worse accuracy, is left out and a reduced ensemble comprising PCA, V-HOG, LG, and *S* is proposed. The performance of the reduced ensemble is optimized with a weighted combination using double sigmoid normalization and only decreases by 0.16% with respect to the full ensemble. Among the remaining classifiers, V-HOG and LG have the greatest DF and correlation rates. However, a reduction of the ensemble by disregarding either LG or V-HOG results in a severe loss of accuracy, as shown in Table 13; the joint performance plummets, respectively, to 97.42% and 96.76%. In turn, removal of symmetry, which has the worst individual performance, results in an accuracy of 97.24%, which is also not affordable. In summary, an ensemble comprising at least PCA+V-HOG+LG+*S* is required to surpass 98% accuracy.

## 5. Discussion

Fusion of classifiers has proven to greatly improve the performance of the individual classifiers. In fact, the descriptors and classifiers presented throughout this chapter have been designed to exploit information of different nature regarding the appearance of the vehicle. As a result, the combination of all the classifiers performs better than any subset of combinations for all image regions, which ratifies the diversity of the sources. Specifically, explicit features have been proven to provide valuable information even if their independent performance is limited. Notwithstanding, some features have been proven to be more diverse than others. Accordingly, reduced ensembles of classifiers have been proposed for each image region, discarding the classifiers that produce little or residual gain. In particular, PCA and log-Gabor features are retained in all the reduced ensembles due to their high diversity. Remarkably, although V-HOG entails better individual performance than PCA, it has higher correlation with LG than the latter; therefore its contribution to the ensemble is smaller, and it is only selected in the reduced ensemble pertaining to the far region. In contrast, symmetry, which is by far the weakest classifier, has proven to provide diverse information and is thus included in the reduced ensemble of three of the four regions. Besides, weighted average combination, especially combined with double-sigmoid normalization, has been shown to be significantly more effective than simple average, as the contributions of the classifiers can be adjusted according to their accuracy and diversity.

Table 15 compares the performance (in terms of accuracy) of the proposed classifier combinations with that of the individual classifiers. Both the full classifier ensembles and the reduced ensembles are referred. We observe that the reduced ensemble boosts the performance of the separate classifiers, even in the front close/middle region, where only two classifiers are utilized. Fusion is especially beneficial in the right and the far regions, which constitute the most challenging scenarios. Indeed, in the right region, the performance of all the individual classifiers is worse than that in the left region owing to the more heterogeneous nature of the traffic participants (slow vehicles, such as buses and trucks). Nevertheless, the combined accuracy is similar to that of the left region. Remarkably, in the far region the performance of the best individual classifiers is boosted in almost 3%, thanks to the classifier fusion, and in fact the achieved accuracy is almost as high as in the other regions.

## 6. Conclusions

In this paper the possibilities for the combination of different sources to achieve vehicle classification through image analysis have been studied. The first part of the study is devoted to the analysis of the individual performance of popular techniques for vehicle verification and the comparison among them. Classifiers based on Gabor and HOG features are shown to achieve the best results and to outperform PCA and other classifiers based on features as symmetry and gradient. However, the outcome of the study discloses that, although these features do achieve high accuracy rates, their performance is limited under some scenarios. Interestingly enough, the performance of Gabor-based classifier fades in the far range, whereas that of HOG-based classifier falls short in the close/middle range, which already points to the necessity of feature combination. In the second part of the study, a methodology for the fusion of classifiers built upon the different features is presented. The experiments reveal that classifier fusion result in a substantial gain of performance, especially in the more challenging scenarios such as the far rage, where it yields a gain of nearly 3% with respect to the best single-feature based classifier.

## Figures and Tables

**Table 1:** Accuracy for the different regions as a function of the principal subspace dimensionality.

	Principal subspace dimensionality							
Region	10	20	30	40	50	60	70	80
Front	93.92	95.80	95.76	96.22	95.38	95.86	95.62	93.70
Left	88.34	89.30	92.12	92.72	93.08	93.32	92.70	92.06
Right	84.30	89.04	90.20	90.72	90.92	91.04	90.88	90.70
Far	89.56	90.44	91.22	91.48	91.52	91.56	91.48	91.44

**Table 2:** Best accuracy results of V-HOG for each image region and their associated parameters (η, β).

V-HOG	Rate	η	β
Front	97.68	4	16
Left	97.02	4	36
Right	95.54	4	16
Far	95.60	4	12

Mean	96.46	4	20

**Table 3:** Accuracy results of log-Gabor filters for the different image regions.

	Front		Left		Right		Far	
	Rate	*F*_0_	Rate	*F*_0_	Rate	*F*_0_	Rate	*F*_0_
Accuracy	98.00	1/2	97.36	1/3	97.06	2/5	91.60	1/3

**Table 4:** Performance of gradient-based descriptor using linear and quadratic Bayesian classifiers.

Region	Linear	Quadratic
Front	94.92	96.94
Left	89.98	91.98
Right	90.94	91.76
Far	88.06	89.22

**Table 5:** Performance of symmetry feature.

	Front	Left	Right	Far
Accuracy	80.18	80.46	78.54	80.50

**Table 6:** Joint probabilities of two classifiers; *a* + *b* + *c* + *d* = 1.

	*D*_*j*_ correct	*D*_*j*_ wrong
*D*_*i*_ correct	*a*	*b*
*D*_*i*_ wrong	*c*	*d*

**Table:** (a)

PCA+V-HOG +LG+*S*+*G*	Accuracy	Recall	Precision
RMM-SA	99.26	99.84	99.68
RMM-WA	99.20	99.72	98.68
DS-SA	99.16	99.42	98.56
DS-WA	99.42	99.76	99.08

**Table:** (b)

PCA+V-HOG + LG+*G*	Accuracy	Recall	Precision
RMM-SA	98.38	99.15	97.60
RMM-WA	98.74	99.51	97.96
DS-SA	98.38	99.15	97.60
DS-WA	99.12	99.64	98.60

**Table:** (c)

PCA+V-HOG+LG	Accuracy	Recall	Precision
RMM-SA	99.02	99.60	98.44
RMM-WA	99.10	99.60	98.60
DS-SA	99.04	99.60	98.48
DS-WA	99.12	99.60	98.64

**Table:** (d)

PCA+LG	Accuracy	Recall	Precision
RMM-SA	98.64	99.51	97.76
RMM-WA	98.88	99.39	98.36
DS-SA	98.58	99.51	97.64
DS-WA	98.94	99.39	98.48

**Table:** (a)

DF	PCA	V-HOG	LG	*S*	*G*
PCA	—	3	2.4	9.8	3
V-HOG	3	—	7.2	7.8	4.6
LG	2.4	7.2	—	2.8	9.2
*S*	9.8	7.8	2.8	—	8
*G*	3	4.6	9.2	8	—

**Table:** (b)

ρ_*i*,*j*_	PCA	V-HOG	LG	*S*	*G*
PCA	1	0.06	0.06	0.03	0.05
V-HOG	0.06	1	0.24	0.03	0.12
LG	0.06	0.24	1	−0.04	0.30
*S*	0.03	0.03	−0.04	1	0.02
*G*	0.05	0.12	0.30	0.02	1

**Table:** (a)

PCA+V-HOG + LG+*S*+*G*	Accuracy	Recall	Precision
RMM-SA	97.62	98.46	96.76
RMM-WA	98.42	98.68	98.16
DS-SA	97.50	98.26	96.72
DS-WA	98.64	98.33	98.96

**Table:** (b)

PCA+V-HOG + LG+*S*	Accuracy	Recall	Precision
RMM-SA	97.78	97.88	97.68
RMM-WA	98.50	98.18	98.84
DS-SA	97.60	97.80	97.40
DS-WA	98.48	97.95	99.04

**Table:** (c)

PCA+V-HOG+LG	Accuracy	Recall	Precision
RMM-SA	98.28	97.79	98.80
RMM-WA	98.22	97.58	98.92
DS-SA	98.28	97.75	98.84
DS-WA	98.26	97.52	99.04

**Table:** (d)

PCA+LG+*S*	Accuracy	Recall	Precision
RMM-SA	95.86	95.52	96.24
RMM-WA	98.36	98.10	98.64
DS-SA	95.30	95.10	95.52
DS-WA	98.30	97.75	98.88

**Table:** (e)

PCA+LG	Accuracy	Recall	Precision
RMM-SA	97.46	97.18	97.76
RMM-WA	97.80	97.13	98.52
DS-SA	97.46	97.18	97.76
DS-WA	97.96	97.21	98.68

**Table:** (a)

DF	PCA	V-HOG	LG	*S*	*G*
PCA	—	8.8	7.2	13.2	14.4
V-HOG	8.8	—	9.2	13.6	16.2
LG	7.2	9.2	—	8	9.4
*S*	13.2	13.6	8	—	21.8
*G*	14.4	16.2	9.4	21.8	—

**Table:** (b)

ρ_*i*,*j*_	PCA	V-HOG	LG	*S*	*G*
PCA	1	0.13	0.13	−0.02	0.12
V-HOG	0.13	1	0.26	0.07	0.25
*LG*	0.13	0.26	1	0.03	0.16
*S*	−0.02	0.07	0.03	1	0.04
*G*	0.12	0.25	0.16	0.04	1

**Table:** (a)

PCA+V-HOG +LG+*S* + *G*	Accuracy	Recall	Precision
RMM-SA	97.64	97.95	97.32
RMM-WA	98.56	98.91	98.20
DS-SA	97.50	97.75	97.24
DS-WA	98.66	98.95	98.36

**Table:** (b)

PCA+LG+*S* + *G*	Accuracy	Recall	Precision
RMM-SA	97.04	97.42	96.64
RMM-WA	98.60	99.11	98.08
DS-SA	96.70	96.99	96.40
DS-WA	98.64	98.95	98.32

**Table:** (c)

PCA+LG+*G*	Accuracy	Recall	Precision
RMM-SA	95.26	95.66	94.84
RMM-WA	98.22	98.36	98.08
DS-SA	94.86	94.96	94.76
DS-WA	98.46	98.52	98.40

**Table:** (d)

PCA+LG+*S*	Accuracy	Recall	Precision
RMM-SA	95.04	95.26	94.80
RMM-WA	98.20	98.63	97.76
DS-SA	94.72	95.01	94.40
DS-WA	98.64	98.96	98.32

**Table:** (e)

PCA+*LG*	Accuracy	Recall	Precision
RMM-SA	97.58	97.38	97.80
RMM-WA	98.18	97.97	98.40
DS-SA	97.58	97.38	97.80
DS-WA	98.32	98.21	98.44

**Table:** (a)

DF	PCA	V-HOG	LG	*S*	*G*
PCA	—	7.60	3.80	15	9.2
V-HOG	7.60	—	13	13.6	17.6
LG	3.80	13	—	8.4	11.2
*S*	15	13.6	8.4	—	22.2
*G*	9.2	17.6	11.2	22.2	—

**Table:** (b)

ρ_*i*,*j*_	PCA	V-HOG	LG	*S*	*G*
PCA	1	0.07	0.02	−0.03	0.03
V-HOG	0.07	1	0.31	0.04	0.23
*LG*	0.02	0.31	1	0.02	0.17
*S*	−0.03	0.04	0.02	1	0.03
*G*	0.03	0.23	0.17	0.03	1

**Table:** (a)

PCA + V-HOG + LG+*S*+*G*	Accuracy	Recall	Precision
RMM-SA	97.16	97.12	97.20
RMM-WA	98.34	98.24	98.44
DS-SA	96.98	96.85	97.12
DS-WA	98.16	98.28	98.04

**Table:** (b)

PCA+V-HOG +LG+*S*	Accuracy	Recall	Precision
RMM-SA	98.38	97.12	95.60
RMM-WA	98.14	98.35	97.92
DS-SA	96.14	96.88	95.36
DS-WA	98.18	98.39	97.96

**Table:** (c)

PCA+V-HOG + *S*	Accuracy	Recall	Precision
RMM-SA	94.52	96.81	92.08
RMM-WA	97.34	98.85	95.80
DS-SA	94.24	96.67	91.64
DS-WA	97.42	98.85	95.96

**Table:** (d)

PCA + LG+*S*	Accuracy	Recall	Precision
RMM-SA	94.52	94.67	94.36
RMM-WA	96.76	95.95	97.64
DS-SA	94.22	94.42	94.00
DS-WA	96.76	95.92	97.68

**Table:** (e)

PCA+V-HOG+LG	Accuracy	Recall	Precision
RMM-SA	97.22	98.05	96.36
RMM-WA	97.26	97.97	96.52
DS-SA	97.24	98.09	96.36
DS-WA	97.24	98.01	96.44

**Table:** (a)

DF	PCA	V-HOG	LG	*S*	*G*
PCA	—	7.8	14.2	21.6	15.4
V-HOG	7.8	—	23.2	13	15.2
LG	14.2	23.2	—	20.2	28.0
*S*	21.6	13	20.2	—	21.2
*G*	15.4	15.2	28.0	21.2	—

**Table:** (b)

ρ_*i*,*j*_	PCA	V-HOG	LG	*S*	*G*
PCA	1	0.04	0.07	0.01	0.05
V-HOG	0.04	1	0.30	0.03	0.14
LG	0.07	0.30	1	0.02	0.21
*S*	0.01	0.03	0.02	1	−0.01
*G*	0.05	0.14	0.21	−0.01	1

**Table 15:** Performance comparison between the proposed fusion scheme and the individual classifiers.

	Full ensemble	Reduced ensemble	PCA	V-HOG	LG	*S*	*G*
Front	99.42	98.94 (PCA+LG)	96.22	97.68	98.00	80.18	96.94
Left	98.64	98.36 (PCA+LG+*S*)	93.32	97.02	97.36	80.46	91.98
Right	98.66	98.64 (PCA+LG+*S*)	91.04	95.54	97.06	78.54	91.76
Far	98.34	98.18 (PCA+V-HOG+LG+*S*)	91.56	95.60	91.60	80.50	89.22
